# Recent advances in gastrointestinal oncology - updates and insights from the 2009 annual meeting of the American Society of Clinical Oncology

**DOI:** 10.1186/1756-8722-3-11

**Published:** 2010-03-23

**Authors:** Milind Javle, Chung-Tsen Hsueh

**Affiliations:** 1 Department of Gastrointestinal Medical Oncology, The University of Texas MD Anderson Cancer Center, Houston, TX 77030, USA; 2 Division of Medical Oncology and Hematology, Loma Linda University, Loma Linda, CA 92354, USA

## Abstract

We have reviewed the pivotal presentations related to gastrointestinal malignancies from 2009 annual meeting of the American Society of Clinical Oncology with the theme of "personalizing cancer care". We have discussed the scientific findings and the impact on practice guidelines and ongoing clinical trials. Adding trastuzumab to chemotherapy improved the survival of patients with advanced gastric cancer overexpressing human epidermal growth factor receptor 2. Gemcitabine plus cisplatin has become a new standard for first-line treatment of advanced biliary cancer. Octreotide LAR significantly lengthened median time to tumor progression compared with placebo in patients with metastatic neuroendocrine tumors of the midgut. Addition of oxaliplatin to fluoropyrimidines for preoperative chemoradiotherapy in patients with stage II or III rectal cancer did not improve local tumor response but increased toxicities. Bevacizumab did not provide additional benefit to chemotherapy in adjuvant chemotherapy for stage II or III colon cancer. In patients with resected stage II colon cancer, recurrence score estimated by multigene RT-PCR assay has been shown to provide additional risk stratification. In stage IV colorectal cancer, data have supported the routine use of prophylactic skin treatment in patients receiving antibody against epidermal growth factor receptor, and the use of upfront chemotherapy as initial management in patients with synchronous metastasis without obstruction or bleeding from the primary site.

## 

The prognosis of advanced gastrointestinal cancers has improved modestly over the last two decades. In the 2009 annual meeting of the American Society of Clinical Oncology (ASCO), it has become clear that targeted therapies and personalized medicine for many cancer types will soon become the standard of care. These data contributed strongly towards the theme of the 2009 meeting - "Personalizing Cancer Care".

### First-line and targeted therapy for advanced gastroesophageal cancer

Human epidermal growth factor receptor 2 (HER2) exhibits tyrosine kinase activity and functions as a growth factor receptor [1]. The overexpression of HER2 as a result of gene amplification has been demonstrated in solid tumors such as breast and gastric cancers, and correlates with aggressive course and poor prognosis [2,3]. Immunohistochemistry (IHC) and fluorescent in-situ hybridization (FISH) are commonly used to measure HER2.

Pre-clinical studies have shown that trastuzumab, a monoclonal antibody against HER2, causes cell cycle arrest at G1 and exhibits antitumor activity in HER2 overexpressed gastric cancer [4,5]. Moreover, trastuzumab can enhance chemotherapeutic efficacy in gastric cancer xenograft with HER2 overexpression, when combined with cytotoxic agents such as capecitabine, cisplatin, or taxane [6]. Phase II studies incorporating trastuzumab with cisplatin-based regimen in patients with advanced gastric cancer overexpressing HER2 have shown encouraging activities [7,8].

The ToGA trial presented at ASCO 2009 screened approximately 3,800 gastric cancer patients from 24 countries [9]. They noted that HER2 expression was detectable in 22% of patients and the concordance rate between IHC and FISH was high at all levels of HER2 positivity [10]. There was a specific pattern of disease which correlated with HER2 expression. Higher rates occurred in intestinal and proximal or gastroesophageal junction cancers than with diffuse or distal gastric cancers. Patients tested positive for HER2 expression were enrolled into a large phase III trial comparing combination of fluoropyrimidine (5-fluorouracil [5-FU] or capecitabine) and cisplatin chemotherapy with or without trastuzumab (Fig. 1). The primary study endpoint was overall survival. The statistics were powered to detect a survival improvement from 10 to 13 months with hazard ratio of 0.77. In the final analysis, median overall survival improved from 11 months with chemotherapy alone, to 13.5 months with the addition of trastuzumab (p = 0.0048). Response rate was 47% in the study arm vs. 34% in the control arm. There were no differences in the rates of congestive heart failure between the two groups although there was a higher rate of asymptomatic decrease in cardiac function in the trastuzumab group. This is not altogether surprising as the median duration of trastuzumab therapy (4.9 months) was shorter than for breast cancer. This study demonstrated that HER2 targeted therapy will be beneficial for 20-25% of gastric cancer cases. The role of trastuzumab as a single agent or as a part of perioperative therapy is worth investigation.

**Figure 1:**
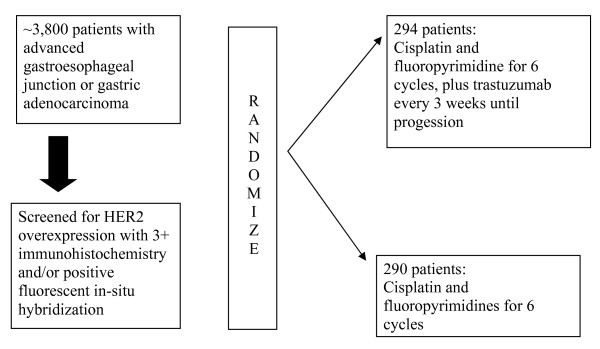
**ToGA study design**.

Other targeted agents being investigated in the phase II setting in gastroesophageal cancer include cetuximab and bevacizumab. Both K-RAS and B-RAF gene mutations are rare in gastric cancer and therefore cetuximab may have an important therapeutic role. The administration of bevacizumab was accompanied with a small risk of bleeding or perforation. However, overall these studies indicate that these combinations are feasible and result in impressive median overall survival rates as compared with historical controls (Table 1; [11-13]).

**Table 1:** Phase II studies incorporating targeted agents in advanced gastroesophageal cancer

Targeted agent	Chemotherapy	N	RR	PFS (months)	OS (months)	Reference
Cetuximab	Irinotecan and FU	49	42%	8.6	16.6	Kanzler et al. [11]

Bevacizumab	Docetaxel, FU and cisplatin	44	67%	12	16.2	Kelsen et al. [12]

Cetuximab	Irinotecan and oxaliplatin	51	60%	6	9.5	Woell et al. [13]

N: patient number; RR: response rate; PFS: progression-free survival; OS: overall survival; FU: 5-fluorouracil

S-1 (TS-1^®^, Taiho Pharmaceutical Co., Ltd.) is an orally active combination of tegafur (a 5-FU prodrug), gimeracil (an inhibitor of dihydropyrimidine dehydrogenase), and oteracil (which inhibits the phosphorylation of 5-FU in the gastrointestinal tract, thereby reducing the gastrointestinal toxic effects of 5-FU) and is widely used in Asia for the management of gastric cancer. Adjuvant therapy with S1 improved survival after gastrectomy and D2 dissection as compared with controls in a prospective trial [14]. FLAGS trial, a phase III and multi-center study, enrolled 1053 patients with advanced gastric cancer, and compared cisplatin with either S-1 or 5-FU as first-line therapy [15]. The primary endpoint was overall survival, and there was no overall survival improvement with the S1-based regimen. However, toxicity was lower in S1 and cisplatin group as compared with cisplatin and 5-FU in a subset analysis, and there was an improvement in survival among patients with the diffuse-type of gastric cancer. Updated findings of JCOG 9912, which was a phase III study conducted in Japan, were reported in 2009 ASCO meeting [16]. This study compared 5-FU, irinotecan plus cisplatin, and S1 as first-line treatment in patients with advanced gastric cancer, and the median survival figures were 10.8, 12.3 and 11.5 months, respectively. Although there was a significant non-inferiority of S-1 to 5-FU (P < 0.001); however, either S-1 or irinotecan plus cisplatin failed to show superiority to 5-FU (P = 0.034 and 0.055, respectively). Based on these results as well as the FLAGS data, it is unlikely that S1 will be developed in upper gastrointestinal cancers in the United States.

### Treatment of localized gastroesophageal cancer

Neoadjuvant chemoradiotherapy (CRT) is commonly used in the United States before esophagecomy for esophageal cancer. The Southwest Oncology Group S0356 study demonstrated that oxaliplatin is safe in the neoadjuvant setting and may potentially replace cisplatin when given with concurrent 5-FU and radiation [17]. Schuhmacher et al. investigated the role of preoperative 5-FU, leucovorin (LV) and cisplatin chemotherapy for operable gastric cancer [18]. Accrual was slow and the study was stopped prematurely after enrolling 144 patients to surgery alone vs. preoperative therapy. There was no significant survival improvement with preoperative chemotherapy, although this resulted in lower margin-positive rate. In European countries, perioperative chemotherapy based on the results of the MAGIC trial with epirubicin, cisplatin and 5-FU is frequently used and this approach will continue to be the standard of care even after 2009 ASCO annual meeting [19].

The Eastern Cooperative Oncology Group (ECOG) E2202 evaluated the safety of a novel form of esophagectomy, called minimally invasive esophagectomy (MIE) in a prospective multi-center trial. MIE involves thoracoscopic and laparoscopic techniques in place of 'open' surgery [20]. As per the E2202 experience, MIE can be performed safely with low post-operative morbidity and mortality.

### Hepatobiliary cancers

The mortality of cholangiocarcinoma is increasing world-wide, particularly in areas with low incidence. Part of this trend may be artifactual, as cancers formerly described as 'liver metastases of unknown primary' are increasingly being classified as intrahepatic cholangiocarcinoma. Gemcitabine or fluoropyrimidines are commonly utilized for the treatment of advanced disease. However, randomized, prospective trial data were lacking in this disease. Valle et al. previously reported the results of a phase II randomized trial of gemcitabine vs. gemcitabine plus cisplatin for the treatment of advanced biliary cancer [21]. In their phase II study, the median time to tumor progression and 6-month progression-free survival (the primary end point) were greater in the gemcitabine and cisplatin arm vs. the gemcitabine-only arm. Based on these results, they initiated a phase III, multicenter trial of the two regimens for advanced biliary cancer (ABC-02) patients, the results of which were reported in ASCO 2009 meeting [22]. Four hundred and ten patients were enrolled (including 149 patients with gall bladder cancer) in a 1:1 randomized trial design. The treatments were well-tolerated in both the arms, surprisingly there was no added toxicity (hematological or grade 3 or 4 non-hematological) despite the addition of cisplatin. There was a significant improvement in survival noted in the gemcitabine plus cisplatin regimen as compared with gemcitabine alone (11 vs. 8 months, p = 0.002). This was also accompanied by an improved progression-free survival. Gemcitabine plus cisplatin therefore becomes a new standard of care for patients with advanced biliary cancer. It is expected that future strategies will add targeted agents to this combination.

BINGO study is a randomized phase II study comparing gemcitabine plus oxaliplatin chemotherapy alone or in combination with cetuximab in patients with advanced biliary cancer [23]. The interim safety analysis of this randomized study indicated no added toxicity with the addition of cetuximab; efficacy data are awaited. Zhu et al. have combined gemcitabine and oxaliplatin with bevacizumab and demonstrated promising efficacy of this combination in advanced biliary cancer [24]. In this study, 18-fluorodeoxyglucose PET scan was used to assess response, and PET responses correlated with survival. Since this disease is often difficult to assess the response by CT scan, further exploration of PET imaging modality is warranted [25].

Transarterial hepatic chemoembolization (TACE) is widely used for the management of regionally advanced hepatocellular carcinoma (HCC). TACE improves local control and is palliative, although its survival impact is controversial. Recently, drug-eluting beads have been employed for TACE in an attempt to increase the control rate. Lencioni et al. presented the results of a randomized trial conducted in 212 patients with unresectable HCC who were randomized to TACE with drug-eluting beads uploaded with doxorubicin vs. conventional TACE with doxorubicin-in-oil emulsion [26]. There was a significant improvement in response rate 52% vs. 44% with the drug-eluting beads, and this was also accompanied by a lower toxicity rate. This therapy is also being investigated in other malignancies such as neuroendocrine tumor and colorectal cancer (CRC) [27,28].

Sorafenib has now become the standard first-line therapy for advanced HCC after the results of the SHARP trial [29]. There are no standard options after progression beyond first-line therapy with sorafenib. Kaseb et al. presented the results of a phase II study of erlotinib and bevacizumab for advanced HCC [30]. Toxicity associated with this regimen was acceptable and the response rate was impressive at 28%. The overall survival was 12 months, and responses were noted in patients who had received prior systemic therapy. Based on these encouraging results, a randomized phase II study of bevacizumab plus erlotinib vs. sorafenib as first-line therapy in patients with advanced HCC is being conducted.

Brivanib is a dual tyrosine kinase inhibitor of vascular endothelial growth factor receptor and fibroblast growth factor receptor. Raoul et al. reported the results of a phase II study of brivanib in 96 patients (38 of them failed prior sorafenib) with advanced HCC [31]. The median survival of patients without prior systemic therapy was 10 months. Anti-tumor effect was noted in patient received no prior systemic therapy and in patients failed prior sorafenib treatment. Fatigue, hyponatremia, diarrhea and hypertension were the important toxicities noted. Randomized phase III studies of first-line treatment comparing brivanib vs. sorafenib, and second-line treatment comparing best supportive care plus brivanib vs. best supportive care with placebo in patients with advanced HCC are ongoing.

### Pancreatic cancer

The study conducted by Gastrointestinal Tumor Study Group in the late 1970s and early 1980s showed that adjuvant therapy with 5-FU plus radiotherapy followed by maintenance 5-FU chemotherapy after surgical resection for pancreatic cancer improved the overall survival [32]. The subsequent ESPAC-1 study suggested that 5-FU chemotherapy was superior to CRT in the adjuvant setting [33]. CONKO-001 study proved that adjuvant gemcitabine was superior to observation [34]. In 2009 ASCO meeting, the results of ESPAC-3, the largest adjuvant study for pancreatic cancer, were presented [35]. This study compared 5-FU plus LV vs. gemcitabine in 1088 patients after curative pancreatectomy, enrolled in 16 countries, mostly in Europe. There was no statistically significant survival difference between these two arms. Thus even in 2009, there appears to be no regimen better than 5-FU as adjuvant therapy in resected pancreatic cancer. The ESPAC group has launched a subsequent phase III study comparing gemcitabine plus capecitabine vs. gemcitabine. In tertiary institutions across the U.S., neoadjuvant therapy is gathering momentum as it appears to limit surgery to those most likely to benefit by excluding the cancers that have aggressive biology. American College of Surgeons Oncology Group has launched a phase II study of neoadjuvant chemotherapy with gemcitabine and erlotinib followed by pancreatectomy and postoperative adjuvant chemotherapy with gemcitabine and erlotinib for patients with operable pancreatic cancer.

Deep vein thrombosis (DVT) is a commonly encountered problem in patients with pancreatic cancer. Pro-thrombotic factors generated by the cancer cells, debility of the patients, dehydration and systemic chemotherapy have been thought to be the attributing factors. DVT in pancreatic cancer patients is associated with a poor prognosis and therefore its prevention is required. The CONKO-004 study randomized 300 patients with pancreatic cancer receiving gemcitabine-based chemotherapy to low-molecular weight heparin (enoxaparin) or observation [36]. The primary endpoint was DVT occurrence in the first 3 months, during which patients received higher dose of enoxaparin (1 mg/kg/day). The secondary endpoint was overall survival. The investigators noted that the incidence of DVT was lower in the treatment arm as compared with the observation arm (10% vs. 1.3%), without any increase in the bleeding risk. However, there was no survival difference between these two arms.

Newer agents that appear to be promising in pancreatic cancer include abraxane, albumin-bound paclitaxel. Von Hoff et al. presented the preliminary efficacy data of the gemcitabine plus abraxane combination for patients with advanced pancreatic cancer [37]. In this study, PET scan was used to measure response. They observed 23% complete metabolic response and more than 60% disease control rate (complete response, partial response and stable disease). The median overall survival was 9 months and there was a correlation between the expression of secreted protein acid rich in cysteine (SPARC) and clinical outcome. This regimen is currently being investigated in a randomized phase III study.

Promising results were also reported with cationic liposomal paclitaxel (EndoTAG-1) in combination with gemcitabine; a median survival of 11.5 months was noted in this study without serious toxicity [38]. These two studies underscore the efficacy of taxanes in this disease and the importance of drug delivery using the nanoparticle formulations. Insulin-like growth factor 1 receptor (IGF1R) targeted antibodies and tyrosine kinase inhibitors are being investigated in a variety of tumor types including pancreatic cancer. Investigators from MD Anderson Cancer Center presented data on genetic variations in the IGF1R pathway and prognosis in locally advanced pancreatic cancer [39]. In their analysis of 105 patients, a clear genotypic profile emerged which correlated with an adverse outcome and could potentially be targeted by IGF1R inhibitors.

### Neuroendocrine tumors

Long-acting somatostatin analogues are widely used for symptomatic, low-grade neuroendocrine tumors such as carcinoids. The survival impact of this therapy was never examined prospectively. The PROMID study randomly assigned patients with inoperable metastatic neuroendocrine tumors with well-differentiated tumor histology to either placebo or octreotide LAR 30 mg intramuscularly every month until tumor progression [40,41]. The primary end point was time to progression, and secondary end points were survival and response rate. The study was conducted in Germany and planned the enrollment of 162 patients. However, enrollment stopped after an interim analysis of 85 patients as the median time to tumor progression in the octreotide LAR group was 15.6 months versus 5.9 months in the placebo group (p = 0.00072) after 6 months of treatment. The most favorable effect was observed in patients with low hepatic tumor load (< 10%) and resected primary tumors. The authors concluded that octreotide LAR significantly lengthens median time to tumor progression compared with placebo in patients with functionally active and inactive metastatic neuroendocrine tumors of the midgut. This study is likely to expand the use of somatostatin analogues to this subgroup of patients although it should be noted that these results are based on a limited cohort of patients for whom no overall survival data is currently available.

### Anal cancer

Squamous cell carcinoma of anus is an uncommon malignancy of lower gastrointestinal tract. Several randomized studies have established CRT with 5-FU and mitomycin-C (MMC) as standard treatment yielding high rates of local control and 5-year disease-free survival without needing surgery or colostomy [42-44]. Due to frequent occurrence of severe toxicities associated with MMC, several phase II studies have used cisplatin instead of MMC in combination with 5-FU and radiotherapy with promising results [45,46]. RTOG 98-11, a US Gastrointestinal Intergroup trial, compared CRT with 5-FU plus MMC vs. neoadjuvant chemotherapy with 5-FU plus cisplatin followed by CRT with 5-FU and cisplatin. In this study, cisplatin-based regimen was shown to be less effective than MMC-based regimen [44]. The main criticism for this study is induction chemotherapy may provide detrimental effect due to delay in starting radiotherapy [47].

James et al. presented a randomized trial of anal cancer from the United Kingdom (ACT II) using 2 × 2 design comparing 5-FU with either cisplatin or MMC during CRT, followed by either observation or two cycles of maintenance chemotherapy with cisplatin and 5-FU [48]. Response to CRT was excellent with about 95% of patients achieving a complete response at 6 months with either MMC or cisplatin-based regimen. Moreover, maintenance chemotherapy didn't affect disease-free or overall survival at three years, with 75% of patients without recurrences whether receiving maintenance chemotherapy or not. Although the rates of non-hematological toxicities were similar between MMC and cisplatin-based regimens, patients receiving MMC-based regimen had more grade 3/4 hematological toxicities (25 vs. 13%, p < 0.001). CRT with MMC and 5-FU remains the standard treatment for anal cancer, and there is no benefit for giving chemotherapy before or after CRT. In circumstances such as shortage of MMC or necessity to avoid severe hematological toxicities, cisplatin may replace MMC for the treatment of anal cancer.

### Rectal cancer

Rectal cancer carries a high chance of local recurrence. Preoperative radiation therapy was compared with preoperative CRT with 5-FU in patients with locally advanced rectal cancer (LARC) including stage II or III rectal cancer, in French FFCD 9203 and European Organization for Research and Treatment of Cancer (EORTC) 22921 studies. Improved local control rate was noted in patients receiving CRT [49,50]. In French FFCD 9203 study, combined treatment led to improved pathologic complete response of 11.4% (vs. 3.6% in the radiation arm) and improved 5-year local failure rates (8.1% vs. 16.5%, respectively). Therefore, neoadjuvant CRT is considered a standard treatment for patients with LARC such as T3 or T4 lesion or with regional lymph node involvement.

Data from phase I to II trials have shown that adding weekly oxaliplatin to 5-FU or capecitabine in preoperative CRT may improve pathologic response with acceptable grade 3/4 toxicities in patients with LARC [51,52]. Three randomized phase III studies, STAR-01 (primary objective: overall survival), ACCORD 12/0405 PRODIGE 2 (primary objective: pathological complete response) and National Surgical Adjuvant Breast and Bowel Project (NSABP) R-04, have been conducted to study the role of oxaliplatin in the neoadjuvant CRT for LARC.

NSABP R-04 by far has the largest target patient number (~1600) with primary objective to compare the rates of local-regional tumor relapse, and has reached more than 80% of the accrual target since July 2004 [53]. The trial was initially designed as a 2-arm study to compare 5-FU vs. capecitabine, and amended in January 2006 to a 2 × 2 design comparing 5-FU vs. capecitabine with/without weekly oxaliplatin as preoperative CRT in patients with LARC.

STAR-01 and ACCORD 12/0405 PRODIGE 2 were presented in 2009 ASCO meeting. The investigational arms in both studies received weekly oxaliplatin to CRT with either 5-FU or capecitabine. As shown in Table 2, both studies showed addition of oxaliplatin to chemoradiotherapy significantly increased grade 3/4 toxicities without affecting local tumor response [54,55]. There were no improvement in the rate of complete responses found at surgery, and no decrease in the number of patients requiring permanent colostomy, when comparing oxaliplatin arm to standard treatment arm. The exploratory analyses have identified reduced incidence of metastatic disease by oxaliplatin in both studies. Longer follow-up is needed to assess the impact on survival endpoints.

**Table 2:** Two phase III studies investigating the role of adding oxaliplatin to preoperative chemoradiotherapy in rectal cancer

Study	Schema	N	Grade 3/4 toxicities (%)	LAR	pCR	Pathological metastatic disease (%)
STAR-01	Radiotherapy (59.4 Gy) and 5-fluorouracil	379	8%	72%	16%	3%
(primary objective:						
overall survival)	Radiotherapy, 5-fluorouracil and oxaliplatin	368	24% (p < 0.0001)	73%	16%	0.5%

ACCORD 12/0405	Radiotherapy (45 Gy) and capecitabine	299	11%	73%	14%	4%
PRODIGE 2 (primary						
objective: pCR)	Radiotherapy, capecitabine and oxaliplatin	299	25% (p < 0.0001)	76%	19%	3%

N: patient number; LAR: low anterior resection; pCR: pathological complete response

### Adjuvant chemotherapy for colon cancer

Monoclonal antibodies directed against vascular endothelial growth factor and epidermal growth factor receptor have been approved to be used in stage IV CRC, but the benefit of these biologic agents in patients with stage II/III disease remains unknown. NSABP C-08 is a phase III randomized study enrolling ~2,700 patients with stage II/III colon cancer after surgery to compare a modified FOLFOX regimen known as mFOLOX6 (every 2 weeks for 12 cycles) vs. bevacizumab and mFOLFOX6 (every 2 weeks for 12 cycles then bevacizumab alone every 2 weeks for 14 cycles) as adjuvant therapy. The safety report was first presented in 2008 ASCO annual meeting, which demonstrated well-balanced grade 4/5 toxicities in each arm [56].

In 2009 ASCO annual meeting, Wolmark et al. presented the efficacy result from this study [57]. Although improvement in disease-free survival (DFS) was observed during the first year in bevacizumab arm (94.3% vs. 90.7%; p = 0.004), the magnitude of this benefit became gradually attenuated with time when patients were no longer on bevacizumab. There was no significant difference in DFS at 3 years (77.4% vs. 75.5% for FOLOFOX group; p = 0.15). Subgroup analysis showed bevacizumab did not improve 3-year DFS for either stage II (~25% of study patients) or stage III.

The companion study of NSABP C-08 is AVANT (BO17920), which is a three-arm, international phase III study in patients with resected stage III or high-risk stage II colon cancer [58]. High-risk stage II disease is defined by any one of the followings: T4 tumor, bowel obstruction or perforation, histological signs of vascular invasion or perineural invasion, age < 50 years, or < 12 lymph nodes analyzed. This study has enrolled ~3450 patients to receive FOLFOX-4 plus bevacizumab or XELOX (capecitabine and oxaliplatin) plus bevacizumab or FOLFOX-4 alone for 24 weeks. Patients in the bevacizumab arms continued to receive bevacizumab for additional 24 weeks, whereas patients in the FOLFOX arm were observed. The primary endpoint of this study is to compare 3-year DFS, and the result is expected to be available in 2010.

North Central Cancer Treatment Group (NCCTG) N0147 and Pan-European Trials in Adjuvant Colon Cancer (PETACC)-8 are two phase III adjuvant studies enrolling patients with resected stage III colon cancer to receive either FOLFOX or FOLFOX plus Cetuximab for 6 months [59]. Since initiation in 2005, there have been more than 4,000 patients all together enrolled in these 2 studies with primary endpoint of comparing 3-year DFS. Both studies have been amended in 2008 after ASCO annual meeting to randomize patients only with wild-type K-RAS tumors. The interim analysis of PETACC-8 is expected in 2011. The pre-planned interim analysis of N0147 has concluded cetuximab plus FOLFOX did not improve 3-year DFS even in patients with wild-type K-RAS tumors. Therefore, N0147 was permanently closed on November 25, 2009, and the cetuximab treatment was discontinued simultaneously.

### Prognostic and predictive biomarkers for stage II colon cancer

Approximately 75-80% of patients with stage II colon cancer are cured with surgery alone, and the benefit of adjuvant chemotherapy is controversial [60]. The International Multicentre Pooled Analysis of B2 Colon Cancer Trials (IMPACT B2) has pooled the stage II populations of five similar randomized trials comparing 5-FU and LV vs. observation. This study included 1,016 patients, and failed to demonstrate any effect of chemotherapy [61]. The United Kingdom QUASAR study which included more than 2,000 patients with stage II colon cancer has shown chemotherapy with 5-FU and LV provided a small improvement (~4%) in rates of recurrence and overall survival (OS) compared to patients on observation [62]. Oncologists have frequently used clinical and pathological features such as tumor stage (T3 vs. T4), tumor perforation, inadequately sampled lymph nodes (<12), poor tumor cell differentiation, and extramural venous invasion, to identify patients who may harbor higher risk for recurrence and potentially benefit from adjuvant chemotherapy [63]. Most of these features are not informative for the majority of patients, and have never been validated in perspective studies.

Emerging data have shown that microsatellite instability (MSI) and chromosome 18q loss of heterozygosity (18qLOH) in colon cancer may be useful as molecular prognostic markers in patients with stage II/III colon cancer [56,64,65]. The ongoing ECOG 5202 study is a perspective study in stage II colon cancer to identify high-risk patients for adjuvant treatment using molecular marker analysis including MSI and 18qLOH [66].

Bertagnolli et al presented 18qLOH analysis from Cancer and Leukemia Group B (CALGB) protocol 9581, which randomized 1738 patients with stage II colon cancer to postoperative treatment with monoclonal antibody 17-1A or observation [67]. The result was initially reported in 2004 ASCO annual meeting. There was no difference in 5-year DFS and OS between patients receiving treatment and on observation. Among these patients, 537 tumor samples were obtained for molecular marker analysis, and 23% had MSI. Of the remaining samples, 101 tumor samples had 18qLOH, and 49 had intact 18q. There were significant differences in OS (98 vs. 85 m) and DFS (92 vs. 78 m) between patients with intact 18q and 18qLOH favoring patients with intact 18q. This result is in consistent with prior report that LOH at 18q is prognostic for DFS and OS in patients with early-stage colon cancer did not receive chemotherapy after surgery [65].

In an effort to develop new clinical tools for risk assessment and treatment decisions in stage II colon cancer, Kerr et al. presented the multi-gene expression assay in patients with stage II colon cancer [68]. This assay is to use real-time RT-PCR to quantitate RNA derived from paraffin-embedded tumor tissue [69]. They have initially identified 761 candidate genes from 1,851 patients' tumor samples in NSABP C-01/C-02/C-04/C-06 and Cleveland Clinic study. After further modeling and analysis, they have prospectively defined the recurrence score based on 7 genes associated with recurrence risk (stromal related genes: FAP, INHBA, BGN; cell-cycle related genes: Ki-67, C-MYC, MYBL-2; and GADD45B). Additionally, 6 genes were chosen and defined as treatment score. The recurrence and treatment scores were validated in 1,436 tumor samples (711 with surgery alone, and 725 with surgery plus adjuvant chemotherapy with 5-FU and LV) from QUASAR study. In 725 patients receiving surgery and adjuvant chemotherapy with 5-FU and LV, treatment score did not predict benefit of adjuvant chemotherapy.

In 711 patients receiving surgery alone, there was significant association between recurrence score and risk of recurrence at 3 years following surgery (P = .004). There were 43.7% in low-risk group (<30 recurrence score), 30.7% in intermediate group and 25.6% in high-risk group (> = 41 recurrence score). Estimated 3-year recurrence risk is 12% (95% CI 9-16%), 18% (95% CI 13-24), and 22% (95% CI 16-29), respectively in low-risk, intermediate and high-risk groups. Multivariate analyses identified three key independent predictors of recurrence in stage II colon cancer after surgery: T4 stage, MSI and recurrence score. In patients with T3 tumor and negative for MSI (~76% of stage II), recurrence score was found to be useful in predicting individual risk of recurrence. This is the first demonstration of a prospectively defined gene expression assay independently predicting risk of recurrence in stage II colon cancer after surgery.

The translational studies of PETACC 3/EORTC 40993/SAKK 60-00 trial were presented in this year's ASCO meeting [70,71]. This study randomized 3,278 patients with stage II or III colon cancer after surgery to 5-FU/LV or 5-FU/LV/irinotecan [72]. There was no significant difference in 5-yr DFS between these 2 treatment arms. Tumor samples were available from 1,404 patients, and MSI was analyzed in 1,327 samples. There was higher incidence of MSI in stage II (22%) vs. stage III (12%). MSI was a significant prognostic factor for relapse-free survival (HR 0.265, p = 0.0044) and overall survival (median follow up 68 months, HR 0.159, p = 0.011) in stage II colon cancer. There was no significant association between prognosis and MSI in stage III colon cancer, and this may be due to small sample size or possible stage specific biological effects. However, MSI was not predictive for the efficacy of irinotecan/5-FU/LV treatment in this study, which differed from the analysis in CALGB 89803 [73]. Both p53 and the SMAD4 genes had prognostic value for stage III but not for stage II colon cancer. Contradictory to previously published report, 18qLOH failed to demonstrate prognostic value in stage II or III colon cancer. These findings suggest that stage II and III colon cancers may differ biologically.

### Metastatic colorectal cancer: Management of skin rash in patients receiving antibody against epidermal growth factor receptor

Skin rash is the most common side effect for patients receiving antibody against epidermal growth factor receptor such as panitumumab. Severe skin rash may delay or interrupt treatment, therefore reducing the effectiveness of treatment. Mitchell et al. presented a randomized study comparing prophylactic skin treatment vs. reactive skin toxicity treatment (treatment after skin rash developed) in 95 patients with metastatic CRC receiving panitumumab-based chemotherapy [74]. Forty-eight patients received prophylactic skin treatment including topically applied sunscreen, moisturizers and corticosteroids with oral antibiotics (doxycycline) starting 24 hours before the first dose of panitumumab, and 47 patients to reactive skin toxicity treatment. Twenty-nine percent of patients in the prophylactic group experienced skin toxicity vs. 62% of those in the reactive treatment group. The incidence of grade 2 or higher skin toxicities was significantly decreased by prophylactic skin treatment. Only 1% of patients in the prophylactic skin treatment arm experienced a dose delay compared with 6% of patients in the reactive skin treatment arm. The data is supportive of the routine use of prophylactic skin treatment in patients receiving epidermal growth factor receptor inhibitors.

### Metastatic colorectal cancer: Upfront chemotherapy in patients with synchronous metastasis

In patients with newly-diagnosed CRC with synchronous metastasis, the benefit of immediate resection of primary tumor in the absence of symptoms (i.e. bleeding, perforation or obstruction) is unclear. Retrospective analyses in the pre-target therapy era have shown that resection of asymptomatic primary tumors was frequently associated with prolonged survival, but was not found to significantly reduce the incidence of life-threatening tumor-related complications [75-77].

Poultsides et al. presented a retrospective reviewed of 233 patients with synchronous metastatic CRC and unresected primary tumors treated with upfront chemotherapy in a single institute [78]. Patients received FOLFOX or irinotecan plus 5-FU and LV with or without bevacizumab as initial treatment. Two hundred seventeen patients (93%) never required surgery to palliate primary tumor related complications. Ten patients (4%) required nonsurgical intervention such as stent or radiotherapy for symptomatic management of the primary site. Neither use of bevacizumab, location of the primary tumor in the rectum, or metastatic disease burden was associated with increased intervention rate. Their findings support the use of upfront chemotherapy as initial management for patients with synchronous stage IV CRC without obstruction or bleeding from the primary site.

The ongoing perspective phase II study, NSABP C-10, will provide more data in the upfront nonsurgical approach [79]. C-10 has been activated since March 2006, and plans to enroll 90 patients with unresectable stage IV colon cancer and synchronous asymptomatic primary tumor. Patients are treated with bevacizumab and FOLFOX without prophylactic resection of the primary tumor. The primary objective is the rate of primary tumor-related events (i.e. obstruction, perforation, fistula, and hemorrhage) that necessitate surgery.

## Summary

We have discussed important presentations in gastrointestinal oncology from 2009 annual meeting of ASCO. The key findings are summarized as the followings, and will lead to paradigm change in clinical practice. Adding trastuzumab to chemotherapy improved the survival of patients with advanced gastric cancer overexpressing HER2. Gemcitabine plus cisplatin has become a new standard for first-line treatment of advanced biliary cancer. Octreotide LAR significantly lengthened median time to tumor progression compared with placebo in patients with metastatic neuroendocrine tumors of the midgut. In patients with resected stage II colon cancer, recurrence score estimated by multigene RT-PCR assay has been shown to provide additional risk stratification. In stage IV CRC, data have supported the routine use of prophylactic skin treatment including oral antibiotics in patients receiving epidermal growth factor receptor antibody, and the use of upfront chemotherapy as initial management in patients with synchronous metastasis without obstruction or bleeding from the primary site.

## Competing interests

The authors declare that they have no competing interests.

## Authors' contributions

Both authors participated in drafting and editing the manuscript. Both authors read and approved the final manuscript.
